# How to detect and reduce potential sources of biases in studies of SARS-CoV-2 and COVID-19

**DOI:** 10.1007/s10654-021-00727-7

**Published:** 2021-02-25

**Authors:** Emma K. Accorsi, Xueting Qiu, Eva Rumpler, Lee Kennedy-Shaffer, Rebecca Kahn, Keya Joshi, Edward Goldstein, Mats J. Stensrud, Rene Niehus, Muge Cevik, Marc Lipsitch

**Affiliations:** 1 Center for Communicable Disease Dynamics, Department of Epidemiology, Harvard T.H. Chan School of Public Health, Boston, MA 02115 USA; 2 Department of Mathematics and Statistics, Vassar College, Poughkeepsie, NY 12604 USA; 3 Department of Mathematics, École Polytechnique Fédérale de Lausanne, Lausanne, Switzerland; 4 Division of Infection and Global Health Research, School of Medicine, University of St Andrews, St Andrews, UK; 5 Specialist Virology Laboratory, Royal Infirmary of Edinburgh, Edinburgh, UK; 6 Regional Infectious Diseases Unit, Western General Hospital, Edinburgh, UK; 7 Department of Immunology and Infectious Diseases, Harvard T.H. Chan School of Public Health, Boston, MA 02115 USA

**Keywords:** Epidemiological biases, Selection bias, Misclassification, Measurement error, COVID-19, Observational data

## Abstract

In response to the coronavirus disease (COVID-19) pandemic, public health scientists have produced a large and rapidly expanding body of literature that aims to answer critical questions, such as the proportion of the population in a geographic area that has been infected; the transmissibility of the virus and factors associated with high infectiousness or susceptibility to infection; which groups are the most at risk of infection, morbidity and mortality; and the degree to which antibodies confer protection to re-infection. Observational studies are subject to a number of different biases, including confounding, selection bias, and measurement error, that may threaten their validity or influence the interpretation of their results. To assist in the critical evaluation of a vast body of literature and contribute to future study design, we outline and propose solutions to biases that can occur across different categories of observational studies of COVID-19. We consider potential biases that could occur in five categories of studies: (1) cross-sectional seroprevalence, (2) longitudinal seroprotection, (3) risk factor studies to inform interventions, (4) studies to estimate the secondary attack rate, and (5) studies that use secondary attack rates to make inferences about infectiousness and susceptibility.

**Supplementary Information:**

The online version of this article (10.1007/s10654-021-00727-7) contains supplementary material, which is available to authorized users.

## Introduction

Since the onset of the coronavirus disease (COVID-19) pandemic, public health scientists have worked tirelessly to provide the knowledge needed to address this new, global crisis. The pandemic has spurred an exceptional number and breadth of scientific studies [1, 2], including epidemiologic ones, with the pace making it both important and challenging for researchers to design and analyze studies in the most robust way possible, and for reviewers and users to accurately evaluate the strength of evidence such studies provide. Using relevant examples from the literature, we discuss potential epidemiological biases arising in various phases of observational studies of COVID-19 and outline possible solutions.

We consider biases arising across five classes of research questions: (1) estimates of seroprevalence [3], (2) estimates of seroprotection [4, 5], (3) studies of risk factors for becoming infected [6], (4) estimates of the secondary attack rate [7], and (5) comparisons of secondary attack rates to make inferences about susceptibility and infectiousness [7].

## Seroprevalence measurement to estimate cumulative incidence

Serological surveillance studies detect SARS-CoV-2 specific antibodies in the population and can provide an estimate of the seroprevalence—the proportion of various groups (e.g., age groups) harboring such antibodies at a single time point or, if repeated, over time. If antibodies are a marker of protection, seroprevalence may provide a direct estimate of the fraction of individuals immune to the virus, although, as we note in the next section, the protective role of antibodies against future infection remains uncertain and may wane over time [8]. If antibodies are a reliable measure of prior infection, then seroprevalence can also be used as a proxy for the cumulative incidence of infection until that time point (more precisely, until an earlier time point, because antibodies take time to rise to detectable levels after infection [8, 9]).

As measures of cumulative incidence, seroprevalence studies can be more accurate than direct counting of case reports, especially for an infection that is often not detected due to limited testing and/or lack of symptoms and where case ascertainment rates have varied drastically over time. Yet to achieve accurate estimates from seroprevalence studies it is important to recruit a representative sample of the population of interest, and to consider measurement error, which can still create bias in a perfectly collected sample. Bias is always in reference to the specific variable which is being estimated in a statistical analysis (i.e., the estimand) so we will suppose we want to estimate the cumulative incidence of infection using seroprevalence among all people living in a particular city. We note the following considerations regarding potential biases in studies where seroprevalence is used to estimate cumulative incidence and use the terms interchangeably below.

**Fig. 1:**
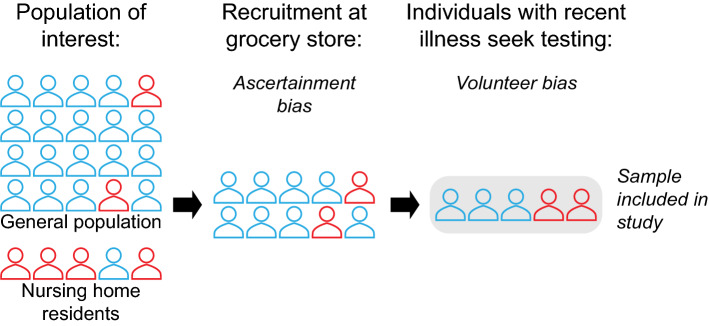
Schematic showing recruitment-based biases in a hypothetical serosurvey. This figure shows a hypothetical serosurvey that aims to measure the underlying seroprevalence in the entire population of a geographic region and performs recruitment among shoppers at a grocery store. Outline color represents prior SARS-CoV-2 infection status (red for prior infection, blue for no prior infection). Ascertainment bias occurs because (1) individuals recruited at the grocery store are likely at slightly higher risk of COVID-19 than average (since individuals who are isolated at home and rarely grocery shop are less likely to be sampled), and (2) nursing home residents and other populations in congregate settings are excluded from the sample. Among individuals present at the grocery store, volunteer bias occurs when individuals who believe they have been infected participate in order to receive testing. Meanwhile, individuals who do not think they have been infected may avoid testing

*Seroprevalence estimates may be unrepresentative of the target population when the individuals enrolled in the study are not representative of that population.* The direction and magnitude of the resulting bias depend on the population for which inference of seroprevalence is being attempted, for example, all residents of a county, and the degree to which the individuals tested diverge from a random sample of that population. Depending on the sampling location and time, people who are present to be sampled may be at higher or lower risk of COVID-19 than average (Fig. 1). Important populations, including those in congregate settings (e.g., nursing homes, prisons), are often excluded. For example, persons residing in long term care facilities (LTCFs) may be over- or underrepresented in serosurveys [10, 11], which depending on their seroprevalence can produce an over or underestimate of true seroprevalence in the population. If persons in LTCFs are not sampled, this can result in a lower seroprevalence estimate for older individuals [11]. For example, the authors of the New York State serosurvey, which recruited a convenience sample at grocery stores across the state, acknowledge that enrollment disproportionately excluded persons from vulnerable groups who may be more likely to self-isolate at home; individuals who died from or were hospitalized or housebound with COVID-19 infection; and individuals living in LTCFs [12]. If researchers hope to generalize inferences to specific risk groups, they must ensure these groups are included in the survey. Once the population of interest has been clearly defined, they should endeavor to randomly recruit individuals from the population of interest and upweight under-sampled groups. Standardization or inverse-probability-of-sampling weighting can mitigate this type of bias, but only if all relevant predictors of seropositivity are included in the correction [13].

*Biases may arise from nonrandom willingness to participate in a survey, even if a random sample of the population is approached to participate.* A serosurvey that successfully reaches the population of interest may still suffer from volunteer bias in who participates in testing. This can bias estimates in either direction. Estimates of seroprevalence will be too high if individuals are more likely to accept testing because they think they have been exposed to SARS-CoV-2. On the other hand, a downward bias will occur if individuals accept testing because they are overcautious or if exposed individuals avoid testing because they do not want a positive test result (Fig. 1). In the study design, volunteer bias can be reduced by sampling from a pre-established cohort with high rates of participation. At least some demographic information should be collected on those who do and do not consent to testing, in order to assess aspects of how representative the consenting population is of those approached. If predictors of non-response are collected, estimates can be corrected in the analysis stage, for example through inverse probability weighting. Serosurveys may want to ask subjects “Do you think you’ve had COVID-19 previously?”, or collect data on symptoms to assess whether volunteer bias is occurring in their sample. Lastly, one innovative approach [14] proposes splitting the survey group into subsets and giving each subset an increasing incentive (for example, money) for participation, enabling researchers to construct a statistical model to predict how seroprevalence would change if everyone participated.

**Fig. 2:**
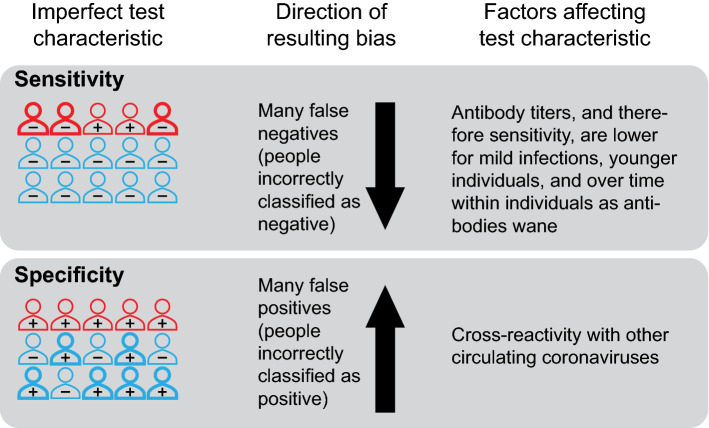
Biases due to misclassification by SARS-CoV-2 antibody tests. The sensitivity of a SARS-CoV-2 antibody test is the probability the test is positive given an individual has been infected with the virus, while the specificity is the probability of a negative test given an individual has not been infected with SARS-CoV-2. Test performance is imperfect; low sensitivity can result in an estimate of cumulative incidence that is too low (as individuals with prior infection are misclassified as negative), and low specificity can result in an estimate of cumulative incidence that is too high (as individuals without prior infection are misclassified as positive). Outline color represents prior SARS-CoV-2 infection status (red for prior infection, blue for no prior infection). The annotation (“+” or “−”) indicates the result of a test for SARS-CoV-2 antibodies. Bold outlines indicate individuals who are misclassified by the test

*False negative serologic tests, if not properly accounted for, can underestimate seroprevalence, while false positive tests, if not properly accounted for, can overestimate it—the latter problem being most serious near the start of the epidemic.* Tests for SARS-CoV-2 antibodies are imperfect. Test performance is described by the test’s sensitivity, which is the ability to identify those who have SARS-CoV-2-specific antibodies, and specificity, which is the ability to identify those who do not have such antibodies. Unless adjusted for in the analysis, the use of imperfectly sensitive tests will underestimate the cumulative incidence of past infections due to the presence of infections not detected by the test (Fig. 2). Conversely, a test with imperfect specificity will incorrectly classify individuals without antibodies as positive, resulting in an overestimate of cumulative incidence if not adjusted for in the analysis (Fig. 2). When a disease is rare, such as COVID-19 early in the pandemic or in areas with low transmission, high test specificity is needed to accurately measure the seroprevalence. For example, the Santa Clara study, which claimed that there were 50–85 times more COVID-19 cases in Santa Clara than previously identified, found 50 individuals positive for antibodies out of 3330 tested [15]; however, the specificity of the test used in that study was uncertain, and a test with 98.5% specificity would be expected to generate 50 false positives on average in that sample if no one had antibodies.

*Adjustments for test sensitivity and specificity should be done with care, accounting for the often small numbers of validation samples and possible differences between the populations in which the tests were validated and the study population.* While most serologic studies do adjust for the test sensitivity and specificity using available estimates for each test, the values of sensitivity and specificity in the study population may be different than in the population used for evaluating test performance, which is commonly made up of hospitalized patients [3]. In particular, sensitivity is often lower for individuals with lower antibody titers. Therefore rates of false negatives are expected to be higher among individuals with less severe disease [8, 16], such as younger individuals [17, 18], individuals who were recently infected and have not yet mounted an antibody response [8, 9], or individuals who were infected long before testing, as antibody titers wane over the weeks and months after infection [8, 9]. For instance, if a seroprevalence estimate among a college student population (where test sensitivity is likely lower) is corrected using a measurement of test sensitivity from older, sicker individuals in validation data then the adjusted estimate will be too low, and vice versa (see [3] for illustrations of this bias through simulation).

*As mentioned above, seroprevalence may underestimate cumulative incidence if some individuals who initially have antibody levels sufficient to test positive on a serologic test have waning levels that drop below the threshold for positivity, a phenomenon sometimes called “seroreversion”*. Low antibody values occur as antibodies are increasing and as they are declining; however, the increase is fast compared to the decline [9, 19], so most individuals with low titers will be those on the decline, except perhaps in a very rapidly growing epidemic, where there will be many very recent infections (e.g., [20] but with antibody titers instead of viral load). Antibodies to seasonal coronaviruses have been shown to decline substantially within a period of a few months to a year [21]. Recent evidence points to the similar disappearance of antibodies to some components of SARS-CoV-2 when data is presented as the percent above a threshold defined as positive [22]. Low sensitivity due to waning antibodies is problematic when using seropositivity as a proxy for the cumulative incidence of infection in a population; for example, the observed temporal decline in seroprevalence in several studies [10, 23], if more than is explainable by sampling variation, likely indicates waning of antibody titers, as the cumulative incidence of infection cannot decrease with time in a closed population. Much remains to be learned (from seroprotection studies) about the nature and duration of protection following infection, so we do not take a position here on whether cumulative incidence and immunity are the same, but we note that it is biologically possible for an individual to be at least partially immune to infection and/or disease due to T cell and B cell memory despite low antibody titers [24–27]. By presenting full distributions of quantitative (e.g., ELISA) values, instead of reporting the percent positive above a threshold, seroprevalence studies can preserve the data for reanalysis as our understanding of antibody kinetics improves.

**Fig. 3:**
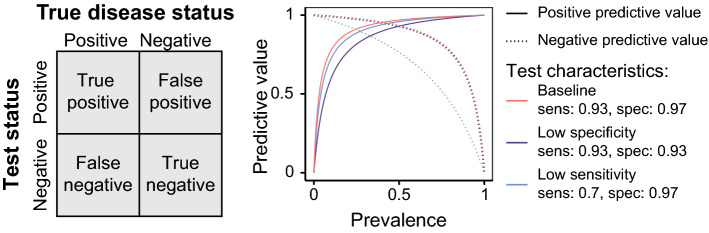
The relative importance of test sensitivity and specificity depends on the underlying seroprevalence in the study population. The value of a test can be described through the positive predictive value (PPV), which is defined as the probability that an individual truly has been infected with the virus given that they test positive and is calculated as the number of true positives divided by the total number of positive tests. Similarly, the negative predictive value (NPV) is defined as the probability that an individual truly has not been infected with the virus given that they test negative and is calculated as the number of true negatives divided by the total number of negative tests. When the underlying seroprevalence is low, test performance is largely a function of specificity, as the majority of individuals in the population have not been infected, while sensitivity is more important as seroprevalence increases. Note that the negative predictive values for the baseline and low specificity tests are very similar so the curves nearly overlap in the figure

Solutions to misclassification include prioritizing high specificity when seroprevalence is low, and high sensitivity when seroprevalence is high (Fig. 3) either through test selection or by using multiple independent tests (e.g., [11]). While estimates of seroprevalence at the population level can potentially be corrected for imperfect test characteristics, this may not remove all sources of bias. As discussed above, bias can remain if, for example, the estimates of test characteristics are obtained from “gold standard” positives and negatives with a different distribution of antibody levels than the true positives and negatives, respectively, in the study population [3]. Additionally, there is uncertainty in the measurement of test specificity and sensitivity, especially for newly developed SARS-CoV-2 antibody tests for which validation datasets may be small. Adjustment using point estimates instead of the full ranges of plausible sensitivity and specificity values underestimates the true uncertainty in seroprevalence estimates. A way to rectify these biases is through the use of a Bayesian approach to adjust seroprevalence estimates for ranges of values for test characteristics [28].

SUMMARY: an ideal study design for SARS-CoV-2 seroprevalence would:

- Use a sample that is representative of the target population. In particular:
  - Recruit participants in a way that does not systematically favor those with unusually high or low levels of exposure.
  - Ask participants and nonparticipants whether they believe they have been infected to detect volunteer bias in a sample.
  - Consider demographics and other information about participants (and ideally also nonparticipants) to facilitate adjustment of results.
- Use an assay for which sensitivity and specificity estimates are available from a population similar to that being studied in terms of disease severity and timing of infections.
- Report the distribution of quantitative (e.g., ELISA) values and not just the percent positive above a threshold to allow analysis of possible seroreversions.
- Adjust seroprevalence estimates for test characteristics, including uncertainty in the measurements of these characteristics.

## Seroprotection

There is evidence to suggest that prior infection with a coronavirus, including SARS-CoV-2, confers some level of immunity and protection against reinfection with the same viral species [21, 29–31]. However, the extent and duration of this protection is unknown, and studies are needed to better characterize immunity to this novel virus. While seroprevalence studies focus on one point in time, seroprotection studies are longitudinal, following people over time to evaluate whether seropositivity confers protection against infection compared to seronegativity. In this case, the causal effect of interest is the direct (biological) effect of seropositivity on future infection. A number of biases can arise in these observational studies.

**Fig. 4:**
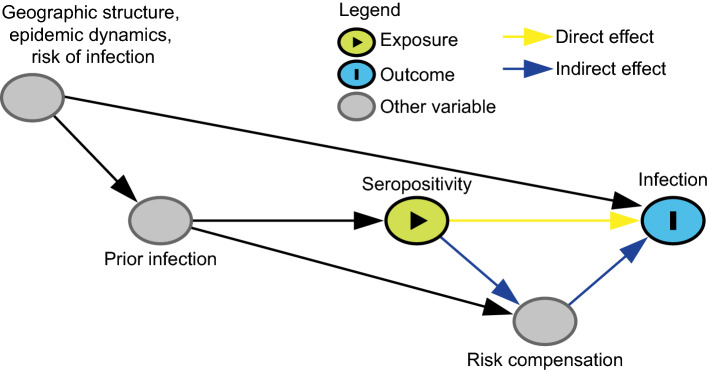
Directed acyclic graph under the alternative hypothesis showing confounding in the estimation of seroprotection. This figure shows the causal relationship between important variables that influence the infection status of an individual. To analyze the effect of seropositivity on the risk of infection, we would need to adjust for geographic structure, epidemic dynamics, the risk of infection and any other variables that are confounders of this exposure-outcome relationship. The effect of seropositivity on infection risk may be mediated by behavior change (induced by knowledge of serostatus) that affects the risk of infection. Disentangling direct (biological) effects of seropositivity and indirect effects through risk compensation is not straight-forward. Geographic structure, epidemic dynamics, and risk of infection are likely or guaranteed confounders of the relationship between seropositivity and future infection. For the purposes of illustrating this particular bias, the directed acyclic graph is drawn under the strong assumption of no additional unmeasured confounding; however, a study of seroprotection, like any observational study, may have other common causes of the exposure (seropositivity) and the outcome (future infection) and it is important to think carefully about additional confounders given unique study settings and designs

*Estimates of the (total) effect of prior infection on (re)-infection may be biased toward the null (no protection) if seropositive individuals remain more exposed to infection than seronegative ones (confounding by risk of infection).* This scenario occurs when there is a confounder that persists through time, such as residing in a crowded household or being an essential worker, that may predict both the exposure (seropositivity) and the outcome (future infection). For example, people in higher-risk occupations are more likely to become infected at each point in time, meaning they are more likely to be seropositive and also more likely to be reinfected (Fig. 4). These positive associations of the persistent confounder with both the exposure and the outcome create a downward bias, causing seropositivity to appear less protective against (or even harmful for) future infection. Limiting the study to groups with high rates of infection and risk of exposure can mitigate this bias while improving power [5], otherwise this bias can be addressed by adjusting for occupation or other factors associated with risk of infection.

*Seroprotection estimates may be biased in either direction if individuals are enrolled at varying phases of their local epidemics or from communities with differently sized outbreaks.* People who are enrolled into a seroprotection study in, for example, the early phase of an epidemic are less likely to be seropositive and have a lower daily hazard of infection than those enrolled during the peak of an epidemic. Similarly, study participants enrolled in communities with lower population infection rates are less likely to be seropositive at enrollment and less likely to become infected after enrollment than study participants from communities with higher population infection rates. Adjustments for day of enrollment and community can reduce this bias [5].

*Imperfect sensitivity or specificity of serologic tests may result in bias toward the null due to misclassification of exposure status (seropositivity) in seroprotection studies.* As noted in the above section on seroprevalence, seropositivity at the population level may imperfectly represent cumulative incidence due to limited sensitivity and specificity and possible changes in these over the course of the antibody response (i.e., declining sensitivity as titers decline). Analogously, at the individual level, which matters for seroprotection studies, seropositivity may be an imperfect representation of an individual's prior infection status. Some of the corrections which are effective at the population level for seroprevalence estimates are not effective at the individual level [32]. Misclassification at the individual level may reduce power as well as cause bias [33].

*Increases in risky behavior by those who are seropositive (risk compensation) may increase the risk of reinfection for such individuals, thereby reducing the magnitude of seroprotection by creating an indirect effect through which prior infection/seropositivity increases the risk of infection*. While this will not bias estimates of the total effect of seroprotection, it will make estimating the direct effect of seropositivity on infection (the effect of interest, which does not include indirect effects mediated by changes in behavior) more challenging; without explicit consideration of behavioral changes, the effects that are evaluated in seroprotection studies will be a combination of the direct and indirect effects (Fig. 4). One potential study design to better isolate the direct effect includes restricting the study to seropositive individuals and comparing high vs. low antibody levels (since people do not usually know this value it should not affect behavior). On the other hand, if antibody levels are a function of previous disease severity [8, 16] and disease severity in turn affects future behavior, this could create a different bias. Moreover, negative control outcomes [34] (e.g., risk of other respiratory infections such as respiratory syncytial virus) could be considered to assess the magnitude of the effect due to behavior differences between seropositive and seronegative individuals. Assuming that SARS-CoV-2 antibodies do not protect against influenza, differences in the number of cases of influenza between SARS-CoV-2 seropositives and seronegatives may indicate behavioral differences between the two groups. Another option may be to perform a formal mediation analysis, but the interventions defining this analysis must be explicit and plausible [35–37].

SUMMARY: an ideal study design for SARS-CoV-2 seroprotection would:

- Explicitly define a causal effect (estimand) of interest, e.g. with respect to a target trial [13].
- Adjust for factors associated with the risk of infection to reduce confounding.
- Control for (in the analysis) or match on (in the study design) time of enrollment and geographic location to mitigate confounding by epidemic dynamics.
- Think carefully about additional confounders given the unique study setting and design.
- Account for, or at least acknowledge, possible bias and/or loss of power due to imperfect sensitivity and specificity of the serologic assay.
- Give thought to the impact of risk compensation among seropositives on the effects estimated in the study.
- Consider the generalizability of the results given the dynamics of the epidemic during the trial.

## Infection risk factors

When a new infectious disease epidemic arises, some of the most important questions are who is most at risk of acquiring infection and, among those most vulnerable to infection, which groups are more likely to face severe illness or death. These questions are especially relevant for COVID-19, as the pandemic has disproportionately affected communities of color, people living in poverty, and other marginalized groups in the United States and internationally [38–41]. Epidemiological studies are crucial for identifying demographic factors (e.g., age, gender, race/ethnicity, disability status, socio-economic status, job type), as well as structural factors (e.g., living and working conditions, literacy, racism, gender inequity) that are associated with the risk of infection in order to inform the allocation of resources and optimize the impact of prevention and treatment interventions [42]. We refer to these as “risk factors” whether they are true causal factors or statistical predictors of infection [43]. Even if we are only looking to identify statistical predictors of infection risk, these studies can still suffer from selection bias due to excluding certain populations or differential testing rates among populations, and from differential misclassification bias due to assuming non-tested individuals are uninfected or by combining test results across test types, timing of tests, and reasons for testing.

**Fig. 5:**
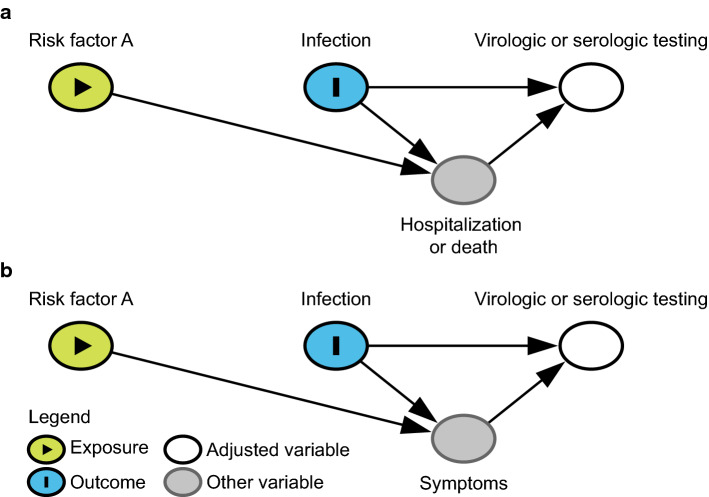
Directed acyclic graph under the null hypothesis showing the possible structure of selection bias due to **a** exclusion from testing and **b** differential likelihood of testing. Under the null hypothesis (of no effect of Risk Factor A on COVID-19 infection) selection bias can be in either direction depending on whether Risk Factor A increases or decreases the likelihood of (**a**) severe disease or (**b**) symptoms among infected individuals. The figures are simplified to illustrate these particular biases so make the strong assumption of no additional unmeasured confounding (i.e., no common causes of any two variables in the figure)

*If a study considers a selected group of individuals who are tested to ascertain infection risk factors, selection bias can play a role (in either direction) if the risk factor of interest is related to the likelihood of hospitalization/death, and hospitalization/death also affects the likelihood of being tested.* For example, if serologic testing is performed in the community (i.e., among non-hospitalized individuals at some point in time during the outbreak), individuals who are currently hospitalized for severe infection or have died because of COVID-19 will not be included in this cohort. If infected individuals with a certain risk factor, such as a comorbidity, are more likely to experience severe disease and become hospitalized or die, there may be a negative correlation between that risk factor and infection in the study because those individuals are underrepresented in the study (Fig. 5a). This will lead to a spurious correlation under the null hypothesis and an attenuated effect estimate if the factor of interest is a true risk factor for infection. This bias can be limited by including individuals who are hospitalized at the time of testing in the sample or accounting separately for their exclusion, as could also be done for individuals who have died due to COVID-19.

*Studies of infection risk factors that include selected groups of individuals who are tested may also suffer from another selection bias if there are differential testing rates or differences in testing criteria between the groups.* For instance, testing for SARS-CoV-2 in many parts of the world has often been limited to suspected cases with symptoms. Thus, symptomatic people are more likely to seek testing or may be more likely to be identified as a contact and tested compared to individuals with atypical or no symptoms. If the risk factor of interest affects the likelihood of such symptoms, then selection bias may occur (Fig. 5b). As another example, suppose a study aimed to investigate the relationship between gender and COVID-19 among tested individuals; women, especially younger women, are more likely to seek medical care than men and therefore are more likely to be tested [44]. Men who receive a test may primarily be those with more severe symptoms or with known exposure to SARS-CoV-2. This selection bias will cause a spurious negative correlation between female gender and infection among those who are tested and enrolled in the study. The bias can be avoided by (1) testing all members of a cohort regardless of symptoms or (2) stratifying on the reason for testing, such as respiratory symptoms [45], although this can limit the generalizability of the results.

**Fig. 6:**
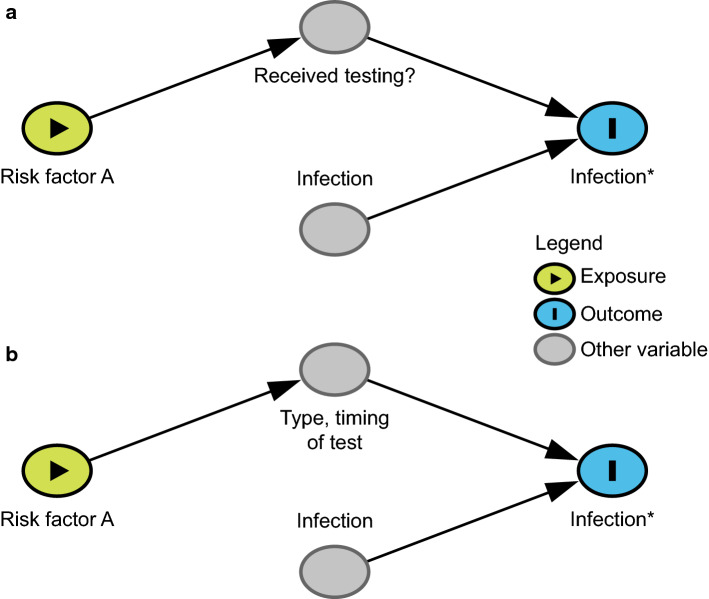
Directed acyclic graph under the null hypothesis showing differential misclassification by **a** whether an individual is tested and **b** the timing or type of test. A study is trying to determine the relationship between Risk Factor A and observed infection status (Infection*), where observed infection status is a proxy for the variable of interest, true infection status (Infection). If (**a**) Risk Factor A influences whether someone is tested and all non-tested individuals are assumed to be uninfected or (**b**) Risk Factor A affects the type of test and timing of testing conducted then under the null hypothesis of no effect of Risk Factor A on COVID-19 rates misclassification can cause upward or downward bias. The figures are simplified to illustrate these particular biases, and therefore make the strong assumption of no additional unmeasured confounding (i.e., no common causes of any two variables in the figure)

*In studies that use confirmed cases among the population as the outcome of interest, including both tested and untested individuals, differential misclassification bias can occur if untested individuals are assumed to be uninfected.* To avoid the selection bias described in the preceding paragraph, studies may include individuals who were not tested for infection and assume they were never infected. Due to this assumption, misclassification of the outcome (infection status) will be much higher among individuals who were not tested (because of lack of symptoms and/or not seeking medical care) compared to tested individuals. This design is unlikely in a formal epidemiological study, but could occur in an ad hoc analysis of case counts. However, this approach can cause differential misclassification bias, in which risk factors for testing appear as risk factors for COVID-19 [46, 47]. Differential misclassification bias will occur if the risk factor increases (or decreases) the chance of testing through a causal pathway unrelated to the probability of infection (Fig. 6a). For instance, suppose a study aims to investigate whether pregnancy is a risk factor for SARS-CoV-2 infection. In some healthcare facilities and municipalities, pregnant women are routinely screened for SARS-CoV-2 infection upon admission for delivery [48]. In this case, pregnancy will be associated with a higher likelihood of being tested and, therefore, a lower likelihood of being misclassified as uninfected due to not receiving testing. This will induce a spurious positive correlation between pregnancy (or factors correlated with pregnancy, such as gender and age) and infection. Similar differential misclassification by whether an individual is tested may also occur with age, as younger individuals are tested less frequently than older individuals, especially in the early phases of the pandemic [49, 50].

*Differential misclassification bias can also arise if testing is performed at different time points, both virologic and serologic testing are used in the outcome measure, or the presence of symptoms affects test performance* [51] (Fig. 6b). For example, suppose a study aims to investigate the relationship between age and SARS-CoV-2 infection risk. Virologic (e.g., PCR) testing of children early in the outbreak was less frequent compared to adults because disease in children is relatively mild [49, 50]. Therefore, children may seem less affected in studies that include only virologic testing. On the other hand, given that serologic testing only became available later [52], children may be more likely to be tested with serologic tests compared to adults in a study that combines both serologic and virologic testing. If the total positivity rates for children and adults were compared, combining both test types, children would appear to have a higher risk of infection under the null because they were more likely to undergo serologic testing (a cumulative vs. point-in-time measure) and it occurred at a later time point. Even in studies using only serologic testing, if children were on average tested later than adults, they would have had more opportunities to become infected, which may still induce a correlation. To prevent this bias, studies of infection risk factors should avoid comparison of serology results from one group with virologic testing from another group, or comparison of combined serologic and virologic testing between groups. Additionally, this bias can be reduced in analysis by adjusting for (e.g., through stratification, matching, or control for) the type and timing (with respect to epidemic time) of the test.

SUMMARY: an ideal study design of SARS-CoV-2 infection risk factors would:

- Test all enrollees using the same test type at a fixed time point or set of fixed time points; and
- Include individuals who were hospitalized or died due to COVID-19 in the enrolled population or account for their exclusion; and
- Enroll individuals who have been randomly selected for testing through an infection surveillance program or some other mechanism; or
- Include only tested individuals (limiting the generalizability) and stratify on or otherwise adjust for the type, timing, and reason for receiving the test, as well as account for individuals not tested due to hospitalization or death.

## Secondary attack rate estimation

We first define and differentiate the terms “infectiousness” and “secondary attack rate” (SAR). We define the term infectiousness as the probability that an infected host transmits the infection to a susceptible person during some well-defined type of contact or interaction. Infectiousness depends on factors associated with the pathogen (e.g., quantity shed, transmission-favoring mutations), host factors in the infected person (e.g., age, symptoms, severity of illness, aerosol generation), and host factors in the susceptible individual (e.g., age, health status). With the accumulation of viral genomes, recent studies have reported evidence of mutations that increase transmissibility. For example, the famous D614G mutation is associated with higher viral load and infection of younger hosts [53], and the SARS-CoV-2 VOC-202012/01 strain with the N501Y mutation has been estimated to have a R_0_ 1.75 times higher than the 501N strain [54]. However, without long-term observation and rich sequencing data we cannot know the relative transmissibility of the virus strains observed in different studies. Therefore, we will limit these discussions to factors relevant for control measures—that is, we assume that the biological features of the virus do not change when comparing different studies. Under this assumption, we expect that infectiousness will differ for different kinds of contact (e.g., being in proximity vs. touching vs. kissing), with different precautions (e.g., with or without mask wearing), and in different environments (e.g., indoor vs. outdoor, degree of ventilation). In an ideal world, infectiousness would be measured by the “susceptible-exposure attack rate”, which is the proportion of exposures per susceptible contact leading to a transmission event—that is, infectiousness per contact, with contact being precisely defined [55]. Since exposures themselves are rarely observed, the SAR is often used as a proxy measure for infectiousness or susceptibility.

The SAR is the proportion of susceptible individuals who become infected within a group of susceptible contacts of a primary (index) case within a given time period [56]. The denominator is the total number of individuals contacted in a particular setting (a particular study may but often does not assess the susceptibility of these individuals at baseline). The numerator is the number of infected secondary cases among those contacts. Contact tracing studies identify and collect information about the index case(s) [usually defined as the first identified infected individual(s)] as well as the close contacts, who are followed to observe the outcome of the exposure. SAR estimation in a particular setting, such as a household or school, can help identify the role of different social interactions, environmental factors, characteristics of the index case, and susceptibility of contacts, which can inform effective strategies to prevent onward transmission. However, biases in study design or data analysis can give rise to inaccurate SAR estimation leading to the mischaracterization of infectiousness or susceptibility. Here we summarize biases that result in inaccurate estimation of the SAR, provide some examples from current studies, and outline recommendations that should be considered to provide accurate estimates and interpretations of infectiousness and susceptibility.

**Fig. 7:**
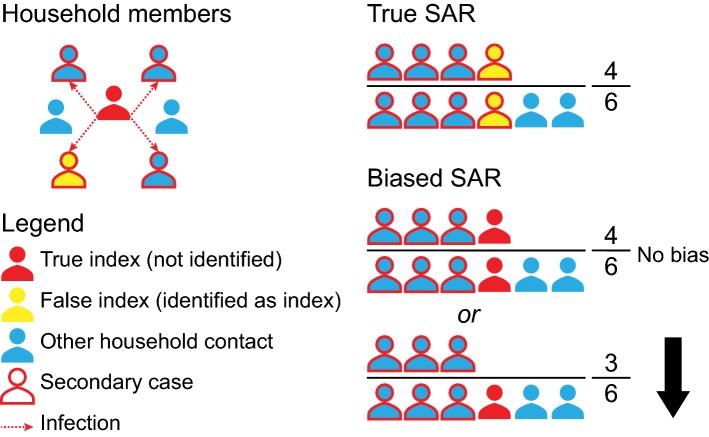
Illustration of index case misclassification where the index and secondary cases are misclassified in a household scenario. In this scenario (top left), each individual has close contacts with every other household member, and the red arrows indicate infections transmitted by the true index case (red individual) to other household members. The true SAR is shown in the top right; the infected contacts of the true index are in the numerator and all contacts are in the denominator. Index case misclassification can happen if one of the secondary cases of the index is falsely identified as the index case (yellow individual). This may cause no bias in the estimation of the SAR value; however, the interpretation of this SAR may be incorrect because we mistakenly attribute the SAR to the false index case, who may have different characteristics, such as age, from the true index case. It can also introduce downward bias if the true index is no longer detected by PCR by the time they are tested (bottom right)

**Fig. 8:**
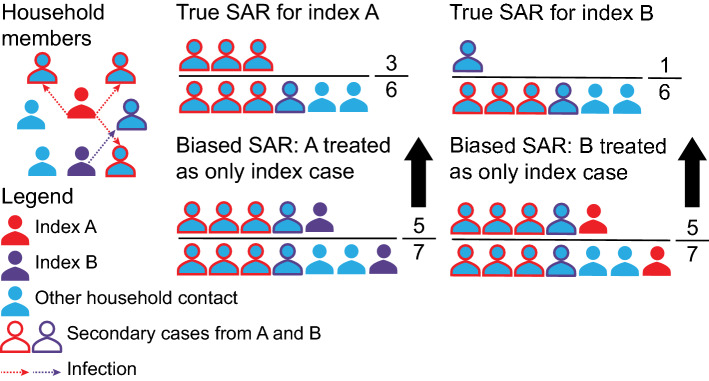
Illustration of index case misclassification when multiple index cases are present but only one is identified as the index case. As shown in the top left, two index cases (red and purple individuals) acquired the infection and transmitted it (red and purple arrows) to other household members. As members of a household are often considered to all be in contact with one another, we cannot distinguish who truly infected whom. The true SARs for each index case are shown in the top. The numerator consists of infected contacts and the denominator consists of all contacts. Upward bias in the SAR can be introduced by falsely attributing all infections, including the other index case, to one of the two index cases

**Fig. 9:**
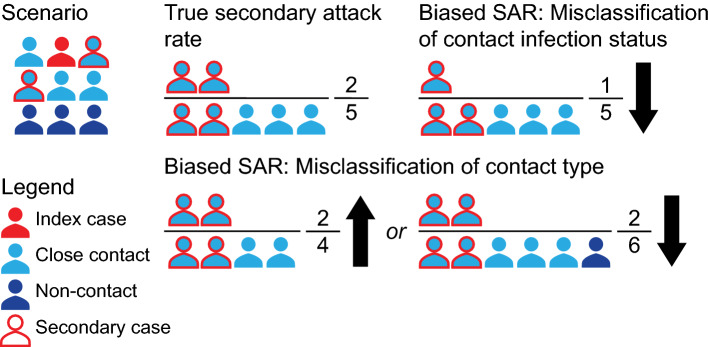
Illustration of misclassification of contact type and contact infection status. As shown in the top left, an infected individual infects some of their close contacts. The true SAR is represented in the top middle; the infected contacts are in the numerator and all close contacts are in the denominator. Bias due to misclassification of contact type can go in both directions. Bias is in the upward direction if some close contacts are missed during contact-tracing (bottom middle), and in the downward direction if non-close contacts are falsely considered as close contacts (bottom right). Misclassification of contact infection status can happen when close contacts are not appropriately tested or followed-up and creates downward bias (top right)

Biases in estimating the SAR can be introduced through two key mechanisms: misclassification of the index case(s) (section “Secondary attack rate estimation: misclassification of the index case(s)”, Figs. 7 and 8, Fig. S1) and misclassification of close contacts (section “Secondary attack rate estimation: misclassification of close contacts”, Fig. 9).

### Secondary attack rate estimation: misclassification of the index case(s)

Misclassification of the index case(s) can happen in two ways: a non-primary (i.e., secondary or tertiary) case is falsely identified as the index case (Fig. 7, Fig. S1) or only one index case is identified when in fact multiple index cases are present (Fig. 8).

*Identifying a non-primary case as the index case may bias SAR estimates up, down, or create no bias in some household settings.* In an outbreak investigation or a household contact tracing study, a secondary case that has more obvious clinical symptoms or epidemiological characteristics (i.e., imported vs. local cases) relative to the true index case may falsely be classified as the index case. For COVID-19, it is possible for an index case to develop symptoms later than the secondary cases [57]; therefore, studies that define the index as the first identified case in a cluster, especially in household context [58, 59], are prone to this error, which could bias the SAR downward or cause no bias (Fig. 7). For scenarios outside the household, the direction of the bias depends on the relative number of contacts from the true index case and the misclassified one (Fig. S1). Epidemiological history can cause an analogous bias; imported cases may be more likely to be identified as index cases than local community cases, especially at a relatively early stage of an outbreak when importation of cases from the epidemic center and local transmissions are both happening simultaneously. For example, identified asymptomatic cases during the third wave in Hong Kong in July and August 2020 were more likely to be imported, which may indicate different screening and testing practices for travelers [60]. This tends to bias the SAR of imported cases upwards and the SAR of local index cases downwards, which may lead to underestimation of existing community transmission or delay the detection of local transmission chains.

*Failure to identify the existence of multiple index cases in a cluster can bias SAR estimates upward* (Fig. 8). If two (or more) cases—A and B—are index cases, one of them may be classified as the only index case and all the secondary cases detected will be attributed to this individual. This causes the SAR to be biased upwards as the secondary cases from B and perhaps even B themselves will be attributed as secondary cases of A. This is most likely in settings with dynamic populations where the source of infection is unclear (i.e., gyms, bars, nursing homes, or gathering events) and multiple index cases may be infecting people at the same time [61, 62]. One example of this bias can be seen in a study from South Korea [58] in which the initial report suggested that the SAR of index cases aged 10–19 years was significantly higher than for index cases aged 20–29, 30–39 and 40–49 years. However, there was a great deal of uncertainty about the true index case in the 10–19 year group due to common sources of exposure with other family members. In a re-analysis [63] of this data, the authors removed household members who potentially shared a common source of exposure with the pediatric cases, resulting in a much lower SAR for the 10–19 year group. Another example is a nursing home outbreak investigation from the Netherlands, where a church service was initially thought to be the source of the outbreak; however, genome sequencing showed multiple clusters in the viral genomes, suggesting multiple introductions to the nursing home [62].

Index case misclassification can be minimized through rigorous follow-up of the selected study population, using frequent and standardized testing, symptom monitoring, and daily contact diaries to track potential infections and index cases. Furthermore, viral genomic analyses, combined with information on contacts, have the potential to more accurately reconstruct transmission pairs or chains of transmission, and further reduce bias in the identification of index cases [64, 65]. In addition, the chain-binomial model can also be used to avoid the misclassification of secondary or tertiary cases as index cases, especially in household settings [66]. An ideal but less practical solution, particularly early in an outbreak, is to conduct a prospective cohort study, either a household study or another population-based study, in which subjects are enrolled before infection and followed up over time.

### Secondary attack rate estimation: misclassification of close contacts

*Imprecise definitions of “close contact” can complicate estimation and interpretation of the SAR.* Ambiguity or bias due to misclassification of close contacts is a common problem in contact tracing studies.

Misclassification of close-contact identification is often related to how the study defines and recruits close contacts, which directly impacts the denominator of the SAR estimate (Fig. 9). If the total number of contacts is not fully documented due to an unclear definition of close contact, it could bias the SAR upward because the closest contacts are more likely to be documented. For example, one study investigated how mask wearing in pre-symptomatic patients can prevent SARS-CoV-2 transmission [67]. In this study, a maximum of ten close contacts of each index case were selected even though the index case may have had more than ten close contacts. This artificial limit would bias the SAR upward for index cases with over ten close contacts. On the other hand, if all contacts including non-close contacts are counted as close contacts, that is, all individuals in the setting rather than well-defined close contacts, it would bias the SAR downward. For example, one study [68] included all employees, family members, and clients of a supermarket over a certain time period, although not all individuals had close contact with the index case. While this study reported separate SARs for different contact groups, the overall SAR reported in this study is not comparable to that of other studies that include only close contacts.

**Fig. 10:**
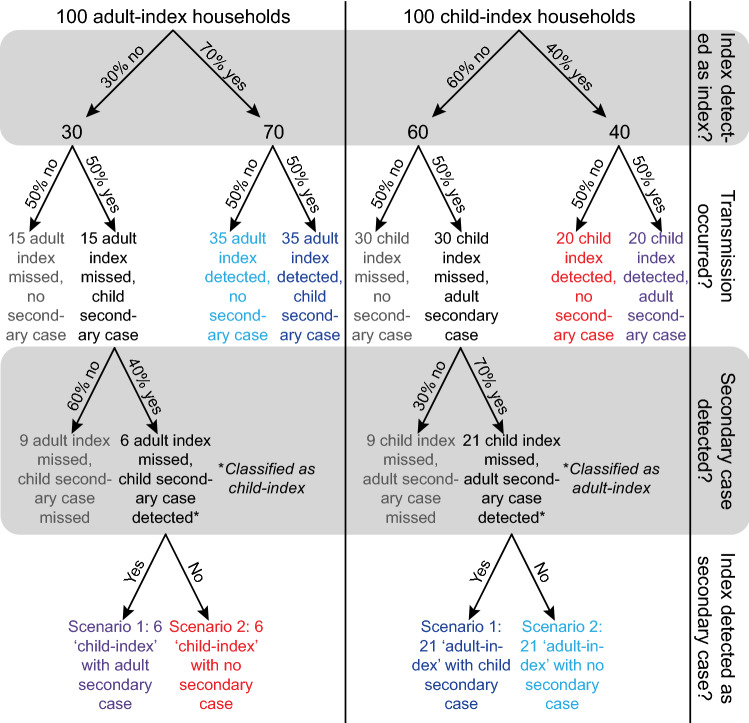
Illustration of differential detection of infection in adults and children. Flowcharts of infection and detection are presented in the diagram. Households shown in the same colors represent the same results, no matter whether they are true or misclassified. Households in grey remain completely undetected. We consider a simplified example for intergenerational household transmission; all households are composed of one adult and one child, so that the only transmission opportunity is to one individual of the other age category. We use 100 households with an adult index case (left column), and 100 households with a child index (right column). This scenario is drawn under the null hypothesis of equal infectiousness of adults and children, and both age groups transmit the infection half (50%) of the time. The only difference between infected adults and children is the probability that they are detected, reflecting differential symptom presentation. We assume that 70% of adults and 40% of children are detected (the numbers chosen are illustrative and the key information is that adults are more likely to be detected). Additionally, we assume that testing works perfectly (i.e., all contacts are tested and identified accurately) and no testing is triggered by contacts outside of the household. We consider two scenarios when an index case is missed and their secondary case is detected. In both scenarios the secondary case is falsely considered to be the index case and the true index is tested as a potential secondary case. In scenario 1, the true index can still be detected and will falsely be considered a secondary case of the false index. In scenario 2, the true index can no longer be detected and the false index will be considered to have not infected anyone. The SARs under both scenarios are calculated in Table 1, which shows that the differential detection of infections in adults and children creates a bias that can go in either direction. Under scenario 1, the SAR is higher for adult indices than for child indices, while for scenario 2 the SAR is higher for child indices than for adult indices

Misclassification of infection status in the close contacts directly affects the numerator of the SAR (Fig. 9). If some secondary infections are missed because contacts are not followed for an adequate duration [68, 69] or different outcome ascertainment procedures are used for different groups [70], it would bias the SAR downward. For example, if only contacts with respiratory symptoms are tested then this could bias the SAR downward as seen in a study that recruited 445 close contacts, but only tested the 54 who developed new or worsening symptoms during active symptom monitoring [70]. This means untested contacts were counted as uninfected. A similar but less severe bias occurs when different tests with imperfect sensitivity are used for contacts, which could also bias the SAR downward. Misclassification may also exist when a study uses real-time polymerase chain reaction (RT-PCR) but tests contacts too early or too late after exposure, resulting in a low yield in test positivity (an example under Fig. 10). For example, RT-PCR missed 36% (95% CI: 28%, 44%) of infected close-contacts, especially among those who were tested in the first few days after exposure [71].

Proposed solutions to avoid misclassification of the identification and infection status of close contacts are to refine study protocols beforehand. For example, a clear close contact definition and a standardized protocol to define and identify all potential close contacts throughout the study can help eliminate misclassification. A standardized and high sensitivity testing plan for all close contacts (ideally, regardless of symptom status), and reasonable follow-up (ideally, at least for the duration of the incubation period, for example, 14 days for COVID-19) to observe the outcome of exposure can provide complete ascertainment of the secondary cases.

### Interpreting comparisons of the secondary attack rate to make inference about relative susceptibility or infectiousness

The previous section cataloged the ways in which various biases could affect SAR estimation. If the SAR can be used to estimate infectiousness, then comparing the SAR between index cases can provide information about the association of index case characteristics (e.g., age) with infectiousness. Likewise, SAR comparisons between contacts of different demographic types can be used to infer the relative susceptibility of different types of individuals. However, there are several factors that need to be considered when using the SAR to infer relative susceptibility or infectiousness. Here we discuss factors that influence the interpretation of the SAR, such as comparing the SAR by characteristics of the index cases or close contacts, contact patterns, and across environmental settings. For demographics, we use adults compared to children as the running example.

If identical biases were present in estimating the SAR for adult and child index cases, we might expect the comparison of infectiousness and/or susceptibility to be unaffected. However, many of the biases depend on, for example, the probability of identifying someone correctly as an index case (as discussed previously in section “Secondary attack rate estimation: misclassification of the index case(s)”), and this may differ between adults and children because children with SARS-CoV-2 infection often have milder or no symptoms [72], and thus may be less likely to be classified as the index case for the cluster than adults. Similarly, children may also be less likely to be identified as secondary cases. Thus, these biases in SAR estimation can differentially affect child and adult index cases and/or contacts, and may lead to biases in comparative infectiousness or susceptibility.

One hypothetical scenario we illustrate here shows how this bias is introduced and the corresponding results, where the bias can go in either direction even under the null hypothesis of equal infectiousness (Fig. 10, Table 1). In this example, where all households have only one child and one adult, the infectiousness of children will be underestimated (and their susceptibility will be overestimated) if missed index cases are misclassified as secondary cases (scenario 1), while the opposite bias will occur if missed index cases are not identified as secondary cases (scenario 2), perhaps because they are no longer positive by the time they are tested. As shown in detail in Fig. 10 and Table 1, differential misclassification between adults and children can lead to biases in either direction in the estimation of their relative infectiousness or susceptibility, even when no such differences exist (i.e., the null hypothesis is true, Fig. 10, Table 1). We use this simple example for illustration; actual studies will typically have a mixture of household structures and may also face the issues described in the next section.

**Table 1:**
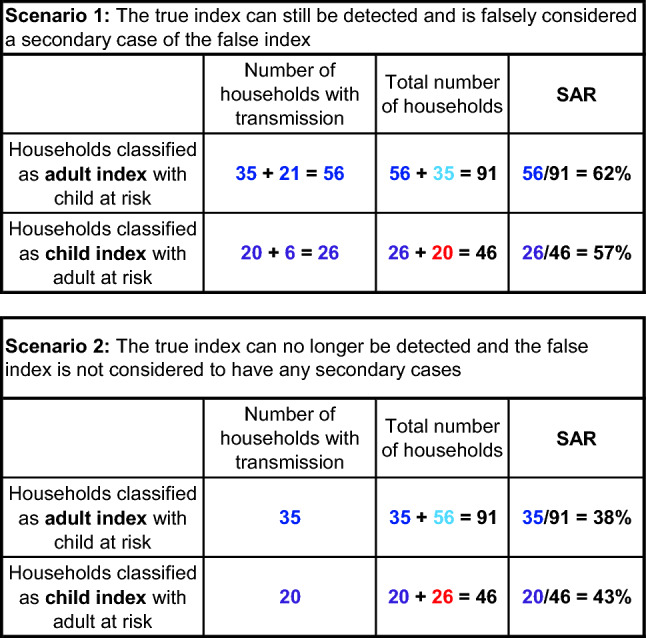
Calculation of the SAR when there is differential detection of infection in adults and children

The tables show the SAR calculation under the two scenarios displayed in Fig. 10. The differential detection of infections in adults and children creates a bias, which can go in either direction. In scenario 1, adult indices have a higher SAR than child indices. In scenario 2, adult indices have a lower SAR than child indices

*Besides factors related to the identification of index cases and close contacts, attention must be paid to contact patterns, including the duration of contact, contact frequency, and the setting where contact occurs, when using the SAR to make inferences about infectiousness.* This includes heterogeneity in contact behaviors, exposure settings, and contact populations. Individuals may infect more secondary cases simply because they have prolonged and closer contacts or riskier behaviors. Higher risks can come from the gathering pattern, such as living together, sleeping in the same room, dining together, or activities such as singing/shouting, and playing board games [73]. For example, spouses have higher attack rates compared to other household contacts with odds ratios of 2.27 (95% CI: 1.22, 4.22) [74] and 3.66 (95% CI: 1.28, 10.5) [75], indicating that the marital status of household contacts should be accounted for in household studies to better disentangle biological from behavioral factors in the infectiousness of household index cases. The setting of the exposure is also important. Different environmental settings, such as indoor vs. outdoor, household vs. non-household [76], environments that tend to generate aerosols (some clinical treatment processes [77]), or poor ventilated settings, may lead to differences in transmission. Suppose there are two types of individuals—type A and type B—who are identical other than the factor being considered. If, all else equal, individuals of type A have prolonged contact with others in a poorly ventilated setting, then type A indices will have more secondary cases than type B indices. Or if type A indices have more contacts in these settings and engage in higher risk activities when the viral load is highest (i.e., -2 to 5 days after symptom onset [78]) and they are more likely to be highly infectious then they may generate more secondary cases (i.e., superspreading events) than type B indices. The third component in contact heterogeneity is the age structure or demographic characteristics of the close contacts. For example, if type A individuals have more contacts with older individuals, then type A indices may end up infecting more people than type B indices, as emerging evidence suggests susceptibility to infection increases somewhat with age [7]. However, studies rarely report the age structure or underlying conditions of close contacts. While this issue can influence studies about infectiousness or susceptibility, it can be easily avoided by collecting relevant information.

Proposed solutions to accurately infer and compare infectiousness include testing stool samples in children because the duration of viral RNA shedding is longer so they are less likely to be misclassified [79, 80]; reporting differences in contact patterns, including activities/behaviors, duration of contact, contact frequency, and contact setting; and collecting detailed epidemiological characteristics of close contacts, such as age, gender, and underlying conditions.

SUMMARY: an ideal study design for SARS-CoV-2 secondary attack rate, infectiousness, and susceptibility estimation would:

- Ensure rigorous follow-up of the study population to minimize misclassification of the index case. This could integrate whole-genome sequencing and phylogenetic analysis to improve the identification of the index case(s), introduction of multiple index cases, transmission directions, chains of transmission, and network interactions. Repeat testing that gives information about viral load may also inform inference of the relative probability that different individuals are index cases [81].
- Use a prospective cohort design, such as a household study, in which subjects are enrolled before infection and followed over time. This could involve frequent serial testing, symptom monitoring, and the use of daily contact diaries. In contact tracing studies, stool samples might be an option for testing children to reduce misclassification of child cases and contacts.
- Clearly define “close contact” and use a standardized protocol for identifying all potential close contacts. Have a standardized and highly sensitive testing plan for assessing infection in all close contacts.
- Use a sufficiently long length of follow-up to observe the outcome of exposure (ideally, at least for the duration of the incubation period, for example, 14 days for COVID-19) among all close contacts.
- Consider hypotheses about shared exposures and collect information on them (for example, travel together to an infected area) [82] or stratify results in main or sensitivity analyses to exclude possible shared exposures [83].
- Simulate in silico outcome data for contact tracing studies under different plausible infection scenarios to understand the impacts of potential misclassifications of index cases or close contacts.
- Be aware of differences in contact patterns, including the duration of contact, contact frequency, contact setting, and epidemiological characteristics of close contacts, when using the SAR to infer that certain groups, such as adults, have higher biological, per-contact infectiousness or susceptibility than other groups, such as children.

## Conclusion

To assist in the evaluation of a continually expanding body of literature on COVID-19, we have outlined and proposed solutions to common biases that can occur across different types of observational studies of COVID-19, including cross-sectional seroprevalence, longitudinal seroprotection, risk factor studies to inform interventions, studies to estimate the secondary attack rate, and studies that use the secondary attack rate to make inferences about relative infectiousness or susceptibility. Across study designs, we identified issues of interpretation, as well as possible biases due to measurement error, selection bias, confounding, and recruitment of non-representative samples. In particular, we highlighted how studies of seroprevalence are subject to misclassification by antibody tests and the possible recruitment of non-representative samples, while studies of seroprotection may suffer from confounding by geographic structure, epidemic dynamics and risk of infection, and their interpretation may be complicated by risk compensation. Studies of infection risk factors may be prone to biased selection of subjects, resulting from the presence of symptoms or hospitalization/death status, and differential misclassification of infection status due to testing factors. Lastly studies of the secondary attack rate can be biased due to misclassification of the index case(s), and failure to correctly identify close contacts and determine their infection statuses, while the use of secondary attack rates to make inferences about infectiousness and susceptibility must be performed carefully with awareness of contact patterns. Although each bias is discussed separately in each study design, multiple biases may coexist and need to be examined carefully in real settings. We hope these thorough descriptions of biases can provide a map or checklist of potential biases to assist with both future study design and the critical interpretation of existing study results.

## Supplementary Information

Below is the link to the electronic supplementary material.

Supplementary Information1 (EPS 1371 kb)
